# The relationship between visceral adiposity index and estimated pulse wave velocity: insights from NHANES database

**DOI:** 10.3389/fnut.2025.1544084

**Published:** 2025-06-11

**Authors:** Yanwei Liu, Shiqiang Yang, Jun Wei

**Affiliations:** ^1^Department of Neurology, The First People’s Hospital of Yibin, Yibin, China; ^2^Department of Neurosurgery, West China Hospital of Sichuan University, Chengdu, China; ^3^Department of Neurosurgery, The First People’s Hospital of Yibin, Yibin, China

**Keywords:** obesity, arterial stiffness, visceral adiposity index, estimated pulse wave velocity, NHANES

## Abstract

**Background:**

Obesity and arterial stiffness are known risk factors for cardiovascular disease, but the relationship between visceral adiposity index (VAI) and estimated pulse wave velocity (ePWV) is still unclear.

**Methods:**

This study used cross-sectional study data from the National Health and Nutrition Examination Survey (NHANES) database from 2007 to 2016. After data cleaning, we performed a comprehensive weighted statistical analysis of the final dataset. This included data on demographics, medical history, test results, and chronic comorbidities. We used restricted cubic spline analysis to explore potential non-linear relationships between VAI and ePWV. We also performed weighted linear univariate and multivariate regression analyses to further explore the relationship between VAI and ePWV. Finally, we performed multiple subgroup analyses and interaction tests, as well as sensitivity analyses, to test the stability of this relationship.

**Results:**

Finally, 10,458 adult participants aged 20 years or older were included in this study, with a mean (SD) age of 49.2 ± 17.4 years after weighted analysis. Restricted cubic spline analysis showed a potentially “inverted-L” non-linear relationship between VAI and ePWV (*P* for non-linearity: <0.001). The inflection point analysis suggests its inflection point is (1.48: 1.38–1.57). Linear multivariate regression analysis suggested a significant positive correlation between VAI (0.2SD) and ePWV values (*β* = 0.1, 95% CI 0.04–0.17, *p* < 0.001). The positive correlation between VAI (0.2SD) and ePWV levels remained stable in model analyses adjusted for all covariates. This association was also consistent across quartiles of VAI (*p*-value <0.001 for trend test). These findings remained stable and consistent in subsequent subgroup and sensitivity analyses.

**Conclusion:**

In this study, we found that elevated VAI values were significantly and positively associated with ePWV, especially below the inflection point of VAI less than 1.48. This association remained robust after adjustment for multiple confounders and was consistent across multiple subgroup analyses. This suggests that abdominal obesity may play an important role in atherosclerosis and highlights the importance of addressing abdominal obesity to reduce cardiovascular risk.

## Introduction

The global burden of cardiovascular disease has increased significantly as a result of economic growth and an ageing population (1). A loss of arterial elasticity with age is often observed. Progressive stiffening of the vessel wall is an inherent physiological feature associated with vascular aging (2). However, this feature is exacerbated by ageing and chronic non-communicable diseases. As an important marker of vascular ageing, arterial stiffness is important in the assessment of vascular health (3). It is also associated with the development of cardiac, cerebrovascular and diabetic diseases. A growing number of studies have confirmed the potential positive association between the degree of atherosclerosis and these diseases (4).

Studies of arterial stiffness are currently a hot topic in the field. The current study has confirmed the diagnostic role of arterial stiffness in the assessment of cardiovascular disease risk (5). Carotid-femoral pulse wave velocity (cf-PWV) is considered the gold standard for assessing aortic stiffness (6). Despite the non-invasive nature of (cf-PWV) and brachial-ankle pulse wave velocity (baPWV) measurements. However, the clinical use of this method is currently extremely limited due to the need for specialised equipment (7). Estimated pulse wave velocity (ePWV) is a new index based on mean blood pressure (MBP) and age that has now been shown to be a reliable predictor of atherosclerosis. Although estimated PWV is not a complete replacement for cf-PWV, recent clinical studies have demonstrated its ability to independently predict cardiovascular disease events and survival (8, 9).

Obesity has been identified as a global epidemiologic challenge and its prevalence continues to rise despite several public health measures currently in place worldwide (10). Recent studies have shown that visceral adipose tissue (VAT) is a more reliable indicator of obesity than total body fat mass (11). There is a potential link between visceral obesity and a variety of conditions, including vascular aging, carotid atherosclerosis, metabolic disorders, and increased all-cause mortality (12, 13). However, imaging techniques such as computed tomography (CT) and magnetic resonance imaging (MRI) are costly, time-consuming, and radiation intensive. There are still major obstacles to universal clinical application. VAI, calculated from waist circumference, body mass index (BMI), triglyceride levels, and high-density lipoprotein cholesterol (HDL-C), has been identified as a gender-specific index. Current studies have demonstrated that VAI is a reliable predictor of a variety of disease states, particularly associated with obesity (14, 15). VAI is a novel index for the assessment of obesity that integrates a variety of metabolic indicators. It can reflect the distribution of visceral fat and metabolic status more comprehensively. ePWV is an important indicator for assessing arterial stiffness. ePWV is closely related to metabolic health and the onset and progression of cardiovascular disease. However, there are relatively few studies on the relationship between VAI and ePWV. The purpose of this study was to analyze the potential relationship between VAI and ePWV using data from a national survey.

## Methods

### Study design and population in NHANES

**Figure 1:**
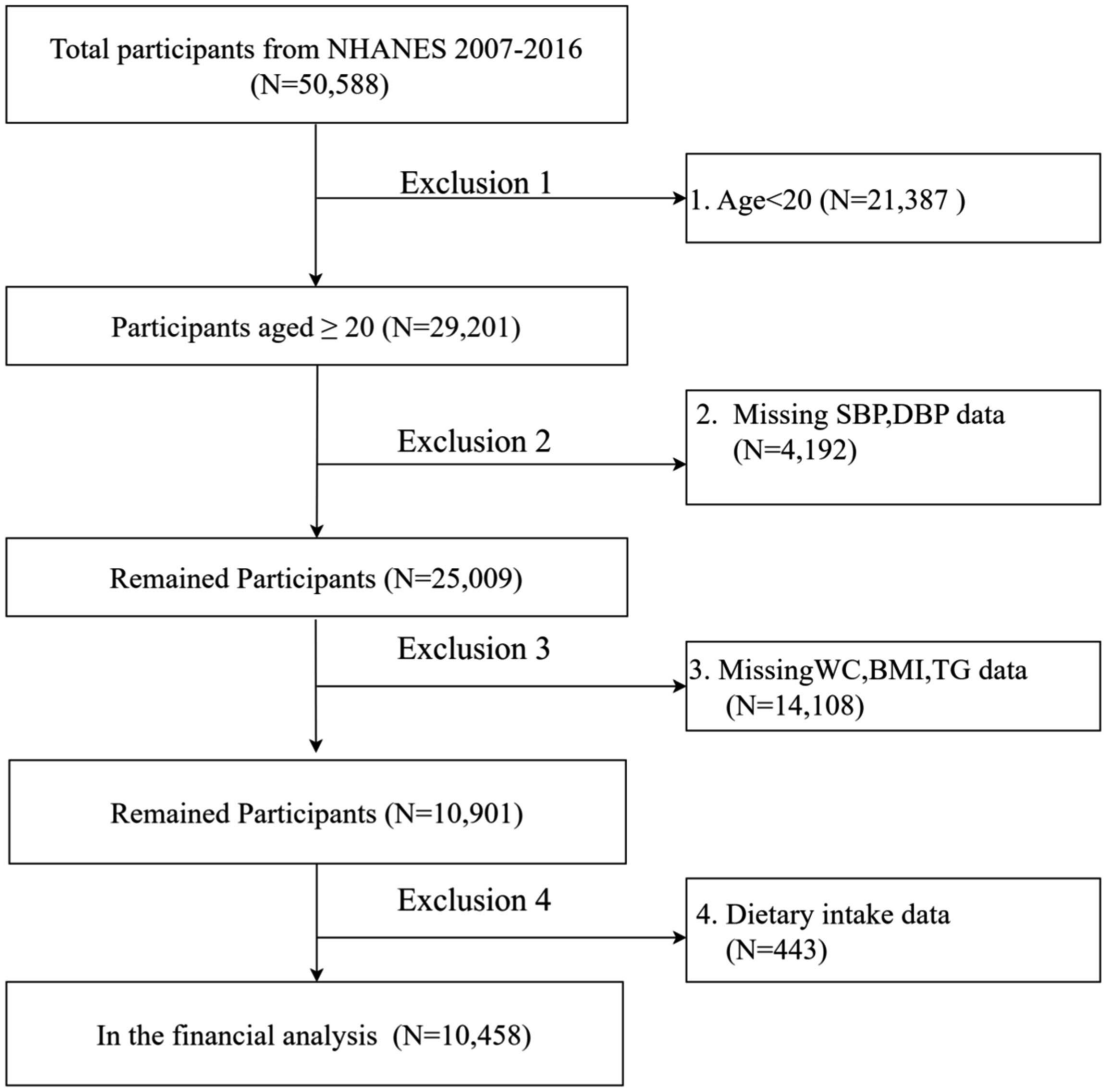
Flowchart of sample selection from NHANES 2007–2016.

This study is based on data from NHANES, a US national survey conducted from 2007 to 2016 using a complex stratified multistage probability sampling design to ensure national representation. The NHANES study protocol was approved by the Research Ethics Review Board of the National Center for Health Statistics, and written informed consent was obtained from all participants. The study report was prepared in accordance with the Enhanced Guidelines for Reporting Observational Studies in Epidemiology. An ethical exemption was granted by the Academic Review Board of the First People’s Hospital of Yibin, as the raw data used in this study were obtained from publicly available databases. This study analyzed data from five NHANES survey cycles (2007–2008, 2009–2010, 2011–2012, 2013–2014 and 2015–2016). And the final analysis data were obtained after strict inclusion and exclusion criteria. The final analysis included 10,458 individuals. (The flow chart is shown in Figure 1).

### Assessment of visceral adiposity index

For men and women, VAI was calculated using two separate equations: for men, VAI = (WC/[39.68 + 1.88 × BMI)] × (TG/1.03) × (1.31/HDL), and for women VAI = [WC/(36.58 + 1.89 × BMI)] × (TG/0.81) × (1.52/HDL), where BMI is body mass index (kg/m2), WC is waist circumference (cm), TG is triglycerides (mmol/L), and HDL is high-density lipoprotein in mmol/L (14).

### VAI normalization process

In the subsequent analyses of this study, we standardized the visceral adiposity index (VAI) to 0.2 standard deviations (SD) to improve the interpretability of the model and the stability of the results. Specifically, we calculated the mean and standard deviation of the VAI and divided each participant’s VAI value minus the mean by 0.2 times the standard deviation. This standardization method is very common in epidemiological and biostatistical studies, especially when dealing with variables with different scales. With this treatment, the regression coefficient can be interpreted as the effect of each 0.2 standard deviation increase in VAI on estimated pulse wave velocity (ePWV).

### Assessment of ePWV

The Arterial Stiffness Reference Values Collaborative Group formulae, as described by Greve et al., were used to calculate ePWV using age (years) and mean blood pressure (MBP, mmHg),as follows: ePWV = 9.587–0.402 × age + 4.560 × 10^−3^ × age^2^-2.621 × 10^−5^ × age^2^ × MBP + 3.176 × 10^−3^ × age ×MBP-1.832 × 10^−2^ × MBP. The formula for MBP is DBP plus 0.4 times the difference between SBP and DBP (16). Blood pressure was recorded by a trained health technician, with participants having three consecutive measurements after 5 min of quiet sitting.

### Covariables data extraction

The selection of covariates was based on previous reports in the literature and clinical experience. The covariates included several demographic factors such as age (in years), sex (categorised as male and female), ethnicity, marital status, poverty income ratio (PIR) and education level. The laboratory results included triglycerides, high-density lipoprotein (HDL), low-density lipoprotein (LDL), glycated haemoglobin (HbA1c) and fasting blood glucose. Dietary information was collected from two 24-h recall interviews (midnight to midnight) in which participants reported the types and amounts of food and beverages (including all types of water) consumed in the 24 h prior to the interview, along with an estimate of energy, protein, carbohydrate, fibre, total fat, and sugar intakes, and information on hypertension, diabetes, coronary heart disease, and stroke. Detailed descriptions of the study variables are available on the official CDC website: www.cdc.gov/nchs/nhanes/. Diagnostic criteria for diabetes mellitus included an official diagnosis by a physician, use of glucose-lowering medication or insulin, a glycated haemoglobin (HbA1c) level of 6.5% or higher, a fasting blood glucose level of 7.0 mmol/L or higher, or a blood glucose level of 11.1 mmol/L or higher on a random blood glucose test or 2-h oral glucose tolerance test. Hypertension was characterised by an average systolic blood pressure of 140 mmHg or higher and an average diastolic blood pressure of 90 mmHg or higher, as diagnosed by a physician or treated with antihypertensive medication.

### Weights processing

To ensure the national representativeness of the study results, we processed sample weights in strict accordance with official NHANES guidelines. As this study integrated NHANES data from five cycles from 2007 to 2016, we used the following weight processing steps:1. weight standardization: sample weights for each cycle were standardized using the formula: standardized weight = original weight/mean of weights for that cycle. 2. weight merging: the standardized weights were merged into a single unified weight using the formula: merged weight = standardized weight/number of cycles. 3. weighted analyses: all statistical analyses were performed using the merged weights to ensure that the results reflected the true picture of the US national population.

### Statistical analysis and sensitivity analysis

The sample was weighted and stratified according to NHANES guidelines, and the NHANES study population was categorised into four different groups based on VAI quartiles. Categorical data were presented as frequencies (expressed as percentages), while continuous variables were presented using the mean and standard deviation if the distribution was normal, and the median and interquartile range (IQR) for skewed distributions. Comparative analyses were performed using one-way analysis of variance (ANOVA) for continuous variables with normal distributions, the Kruskal-Wallis H test for continuous variables with skewed distributions, and the chi-squared test for categorical variables to assess statistical significance between different groups.

Prior to linear regression analysis, we performed an initial test of the distribution of the dependent variable (estimated pulse wave velocity, ePWV). Although the original distribution of ePWV may not be exactly normal, the key assumption of the linear regression model is that the residuals should be approximately normal. Therefore, the distribution of the residuals was tested as follows: histograms of the residuals were plotted and the Shapiro–Wilk normality test was performed. The results show that the distribution of the residuals is close to normal and the *p*-value of the Shapiro–Wilk test is greater than 0.05, indicating that there is not enough evidence to reject the hypothesis that the residuals are normally distributed. We also attempted to log-transform the ePWV to improve the normality of its distribution, but ultimately decided to use the original ePWV values for analysis because the interpretation of the log-transformed values was not as intuitive as the original values. We confirmed the appropriateness of using the raw ePWV values for linear regression analysis using residual tests.

In this investigation, potential confounding variables were first identified through a screening process, and then a series of statistical analyses were performed to explore the relationship between VAI and ePWV. In this study, the restricted cubic spline method was used to model the exposure-outcome relationship. Where potential non-linear correlations were detected, inflection point analyses were performed to identify inflection points where the relationship between VAI and ePWV changed significantly. Univariate and multivariate linear regression analyses and model analyses with stepwise adjustment for covariates were then performed to further explore the potential relationship between VAI and ePWV. For modelling analyses, we compared VAI when it was used as a continuous or categorical variable. To assess the relationship between VAI and ePWV, we used weighted multivariate linear regression analyses with stepwise adjustment for multiple potential confounders. We developed four different models with progressively more adjusted variables. Model 1 did not adjust for any confounders and assessed only the crude correlation between VAI and ePWV; model 2 adjusted for sex and race; model 3 further adjusted for education level and PIR based on model 2; and model 4 further adjusted for other confounders such as hypertension, diabetes mellitus, cardiovascular disease, SBP, DBP, cigarette smoking and alcohol consumption based on model 3. In addition, we examined the interactions between the main confounders and VAI and performed several sensitivity analyses to ensure the robustness of the results. We also performed detailed diagnostic analyses of the final model, including residual analysis, linear hypothesis testing, and multiple covariance testing, to ensure the applicability of the model and the reliability of the results.

For the statistical computations, the Free Statistics software version 2.0 (Beijing, China) and the R programming environment version 4.3.2 were utilized. The *p*-values reported are two-tailed, with a threshold of less than 0.05 set to determine statistical significance, thereby allowing for the identification of associations that are unlikely to occur by chance.

## Results

### Study population

This study analyzed data from five NHANES survey cycles (2007–2008, 2009–2010, 2011–2012, 2013–2014, and 2015–2016), initially excluding participants younger than 20 years (*n* = 21,387) from the total of 50,588 individuals. We then excluded participants with missing systolic blood pressure data (*n* = 4,129), diastolic blood pressure data (*n* = 63), waist circumference data (*n* = 1,142), body mass index data (*n* = 55), triglyceride data (*n* = 12,911), and other variables (*n* = 443). With strict exclusion, the missing covariate data for the remaining participants was less than 5%. As a result, multiple interpolation was used to cleanse the study, resulting in the inclusion of 10,458 subjects in the final analysis (Figure 1).

### Characteristics of the participants

All participants were divided into four groups according to baseline VAI quartiles. As shown in Table 1, group Q1 (VAI: <0.87, *N* = 2,575); group Q2 (VAI: 0.87–1.42, *N* = 2,642); group Q3 (VAI: 1.42–2.41, *N* = 2,608); and group Q4 (VAI: >2.41, *N* = 2,633). Data on demographic characteristics, dietary intake, examination data, laboratory data, and questionnaires for all participants in each subgroup are shown in Table 1.

**Table 1:** Baseline characteristics of participants in NHANES (*N* = 10,458).

Variables	Total (*n* = 10,458)	Quartile1 (*n* = 2,575)	Quartile2 (*n* = 2,642)	Quartile3 (*n* = 2,608)	Quartile4 (*n* = 2,633)	*p*
Demographics data						
Gender, *n* (%)						< 0.001
Male	5,192 (49.6)	1,368 (53.1)	1,292 (48.9)	1,254 (48.1)	1,278 (48.5)	
Female	5,266 (50.4)	1,207 (46.9)	1,350 (51.1)	1,354 (51.9)	1,355 (51.5)	
Age, year	49.2 ± 17.4	46.1 ± 17.8	48.7 ± 17.8	50.8 ± 17.3	51.3 ± 16.3	< 0.001
Race/Ethnicity, *n* (%)						< 0.001
Mexican American	1,658 (15.9)	258 (10)	384 (14.5)	489 (18.8)	527 (20)	
Non-Hispanic White	1,200 (11.5)	221 (8.6)	302 (11.4)	332 (12.7)	345 (13.1)	
Non-Hispanic Black	4,578 (43.8)	1,030 (40)	1,131 (42.8)	1,122 (43)	1,295 (49.2)	
Other Hispanic	1989 (19.0)	771 (29.9)	569 (21.5)	412 (15.8)	237 (9)	
Other Race	1,033 (9.9)	295 (11.5)	256 (9.7)	253 (9.7)	229 (8.7)	
Education, *n* (%)						< 0.001
<9th Grade	1,134 (10.8)	182 (7.1)	254 (9.6)	308 (11.8)	390 (14.8)	
9th-11th Grade	1,512 (14.5)	320 (12.4)	354 (13.4)	394 (15.1)	444 (16.9)	
High school graduate	2,347 (22.4)	521 (20.2)	611 (23.1)	603 (23.1)	612 (23.2)	
Some college	2,988 (28.6)	751 (29.2)	733 (27.7)	740 (28.4)	764 (29)	
≥College graduate	2,477 (23.7)	801 (31.1)	690 (26.1)	563 (21.6)	423 (16.1)	
Marital Status, *n* (%)						< 0.001
Married	6,345 (60.7)	1,469 (57)	1,579 (59.8)	1,643 (63)	1,654 (62.8)	
Divorced	4,113 (39.3)	1,106 (43)	1,063 (40.2)	965 (37)	979 (37.2)	
PIR, Mean ± SD						< 0.001
Low income (<1.3)	3,093 (32.2)	674 (28.4)	728 (29.9)	762 (31.9)	929 (38.5)	
Medium (1.3–3.5)	3,604 (37.5)	888 (37.4)	912 (37.5)	935 (39.2)	869 (36)	
High income (≥3.5)	2,906 (30.3)	810 (34.1)	794 (32.6)	689 (28.9)	613 (25.4)	
Dietary data						
Energy, kcal	1938.0 (1434.0, 2570.0)	2011.0 (1488.0, 2660.5)	1934.0 (1432.0, 2552.0)	1906.5 (1417.0, 2539.2)	1892.0 (1387.0, 2537.0)	< 0.001
Protein, gm	74.5 (52.8, 100.9)	77.1 (55.3, 106.7)	75.0 (52.8, 100.4)	72.6 (52.3, 98.7)	73.3 (51.0, 99.0)	< 0.001
Carbohydrate, gm	232.6 (167.8, 312.8)	231.7 (166.5, 314.2)	231.3 (168.2, 311.4)	234.0 (169.2, 312.5)	232.8 (167.2, 313.0)	0.877
Total sugars, gm	95.3 (58.2, 143.7)	93.4 (55.7, 142.6)	95.6 (59.7, 140.3)	96.6 (59.6, 144.7)	95.4 (58.2, 147.5)	0.118
Dietary fiber, gm	14.6 (9.6, 21.6)	14.9 (9.9, 21.6)	14.7 (9.8, 21.5)	15.0 (9.7, 21.7)	14.1 (9.2, 21.4)	0.019
Total fat, gm	71.3 (47.4, 102.0)	74.8 (51.2, 108.0)	71.8 (47.4, 100.6)	70.8 (46.9, 101.1)	68.0 (44.6, 98.5)	< 0.001
Examination data						
BMI, kg/m^2^	28.9 ± 6.6	25.9 ± 5.8	28.3 ± 6.4	30.1 ± 6.5	31.3 ± 6.3	< 0.001
Wait circumference, cm	99.1 ± 16.0	90.5 ± 14.4	97.3 ± 15.3	102.4 ± 15.2	106.2 ± 14.6	< 0.001
SBP, mmHg	122.8 ± 17.7	120.1 ± 17.5	122.1 ± 17.7	123.2 ± 17.5	125.6 ± 17.7	< 0.001
DBP, mmHg	69.0 ± 12.0	67.8 ± 11.6	68.5 ± 11.8	69.1 ± 12.1	70.5 ± 12.4	< 0.001
Laboratory data						
Hemoglobin A1c (%)	5.8 ± 1.1	5.5 ± 0.7	5.6 ± 0.9	5.8 ± 1.1	6.1 ± 1.4	< 0.001
FPG, mmol/L	6.1 ± 2.0	5.6 ± 1.2	5.8 ± 1.5	6.1 ± 2.0	6.7 ± 2.7	< 0.001
TG, mmol/L	1.2 (0.8, 1.7)	0.6 (0.5, 0.8)	1.0 (0.8, 1.1)	1.4 (1.2, 1.6)	2.2 (1.8, 2.9)	< 0.001
LDL-cholesterol, mmol/L	2.9 (2.3, 3.5)	2.6 (2.1, 3.2)	2.9 (2.3, 3.5)	3.0 (2.5, 3.6)	3.1 (2.4, 3.8)	< 0.001
HDL-Cholesterol, mmol/L	1.3 (1.1, 1.6)	1.7 (1.4, 2.0)	1.4 (1.2, 1.7)	1.2 (1.1, 1.4)	1.0 (0.9, 1.2)	< 0.001
Questionnaire data						
Hypertension, *n* (%) Yes	3,738 (35.7)	680 (26.4)	891 (33.7)	978 (37.5)	1,189 (45.2)	< 0.001
Diabetes, *n* (%)Yes	1,293 (12.4)	166 (6.4)	255 (9.7)	378 (14.5)	494 (18.8)	< 0.001
CVD, *n* (%)Yes	419 (4.0)	81 (3.1)	90 (3.4)	103 (3.9)	145 (5.5)	< 0.001
Secondary calculated data						
MAP, mmHg	90.5 ± 11.7	88.7 ± 11.6	89.9 ± 11.5	90.7 ± 11.7	92.5 ± 11.7	< 0.001
ePWV, m/s	7.7 (6.4, 9.8)	7.1 (6.2, 9.3)	7.6 (6.4, 9.7)	8.0 (6.6, 10.0)	8.1 (6.8, 9.9)	< 0.001

BMI, body mass index; PIR, family poverty income ratio; SBP,systolic blood pressure; DBP: diastolic blood pressure;, FBG: fasting blood glucose; TG, Triglyceride; CVD, Cardiovascular disease; MAP,mean arterial pressure;ePWV, Estimated pulse wave velocity; Quartile 1, VAI<0.87; Quartile 2, VAI(0.87–1.42); Quartile 3, VAI(1.42–2.41); Quartile 4, VAI>2.41. All statistical analyses in table were weighted using sample weights provided by NHANES to ensure that the results are nationally representative.

### Univariate and multivariate linear regression analyses

In this study, univariate regression analysis between VAI and various covariates and ePWV was systematically analysed using linear regression analysis. The results indicated a possible positive association between VAI and ePWV [*β*: 0.1; (95% CI: 0.04, 0.17), *p* = 0.003]. In addition, univariate analyses performed with all covariates for exposure suggested statistically significant differences between age, male, race, education, PIR and energy intake and outcomes (all *p* < 0.001). Detailed results are presented in Supplementary Table 1.

Covariance tests for covariates were then performed using methods such as variance inflation factor (VIF). The results indicated a significant colinearity between energy intake, carbohydrate intake, total sugar intake, total fat intake, BMI, WC, TC, TG, HDL-c and LDL-c. Detailed results are presented in Supplementary Table 2. Covariates that were significantly colinearity were removed in subsequent multivariate analyses.

In the multivariate linear regression analysis, we found a potential positive association between VAI and ePWV. This association remained significant after adjustment for multiple confounders. In model 1, no adjustment for covariates was made and the results indicated a possible positive correlation between VAI and ePWV [*β*: 0.1; (95% CI: 0.04, 0.17), *p* = 0.003]. Stable positive associations were also observed in models with stepwise adjustment for covariates. In model 4, adjusted for all covariates, the results were suggestive [*β*: 0.09; (95% CI: 0.02, 0.16), *p* = 0.007]. All participants were then divided into four different groups according to the quartiles of the VAI. The lowest group (Q1, VAI < 0.87) was used as the reference group. Linear multivariate regression analysis continued with four different models. No covariate adjustment was performed in model 1. Groups Q2 [*β*: 0.31; (95% CI: 0.19, 0.43), *p* < 0.001], Q3 [β: 0.54; (95% CI: 0.42, 0.65), *p* < 0.001] and Q4 [β: 0.62; (95% CI: 0.50, 0.74), *p* < 0.001] showed a progressively higher positive correlation. The results remained stable and consistent in model 4 adjusted for all covariates. The results remained stable and consistent in models with stepwise adjustment for covariates. In model 4 adjusted for all covariates, the results were Q2 [β: 0.27; (95% CI: 0.16, 0.38), *p* < 0.001], Q3 [β: 0.49; (95% CI: 0.37, 0.60), *p* < 0.001] and Q4 [β: 0.53; (95% CI: 0.41, 0.64), *p* < 0.001]. Detailed results are presented in Table 2. We also tested for interactions between the main confounders and VAI and found no significant interactions. The results of the sensitivity analysis were consistent with those of the main analysis, further validating the robustness of the results. Model diagnostic analysis showed that the residual distribution was close to normal, the linearity assumption was valid, and no significant multicollinearity problems were found.

**Table 2:** Linear multiple regression analysis model to assess the relationship between visceral adiposity indexs and estimated pulse wave velocity concentrations.

Variable	Model 1		Model 2		Model 3		Model 4	
	Crude. Coefficient (95CI)	*p*-value	Adjusted. Coefficient (95CI)	*p*-value	Adjusted. Coefficient (95CI)	*p*-value	Adjusted. Coefficient (95CI)	*p*-value
VAI (0.2SD)	0.1 (0.04,0.17)	0.003	0.1 (0.03 ~ 0.17)	0.004	0.09 (0.02 ~ 0.17)	0.009	0.09 (0.02 ~ 0.16)	0.007
Quartile1 (<0.87)	0(Ref)		0(Ref)		0(Ref)		0(Ref)	
Quartile2 (0.87–1.42)	0.31 (0.19 ~ 0.43)	<0.001	0.34 (0.22 ~ 0.46)	<0.001	0.3 (0.19 ~ 0.42)	<0.001	0.27 (0.16 ~ 0.38)	<0.001
Quartile3 (1.42–2.41)	0.54 (0.42 ~ 0.65)	<0.001	0.6 1 (0.48 ~ 0.72)	<0.001	0.53 (0.41 ~ 0.65)	<0.001	0.49 (0.37 ~ 0.6)	<0.001
Quartile4 (>2.41)	0.62 (0.5 ~ 0.74)	<0.001	0.67 (0.56 ~ 0.79)	<0.001	0.61 (0.48 ~ 0.72)	<0.001	0.53 (0.41 ~ 0.64)	<0.001
Trend.test		<0.001		<0.001		<0.001		<0.001

CI, Confidence Interval; PIR, Poverty income ratio; Model 1: unadjusted; Model 2: adjusted for gender+race; Model 3: adjusted for Model 2 + (educational level and PIR); Model 4:adjusted for Model3 + (Hypertension, Diabetes, Cardiovascular disease, Systolic blood pressure, Diastolic blood pressure, Smoking and Alcohol consumption).

### Restricted cubic spline models

**Figure 2:**
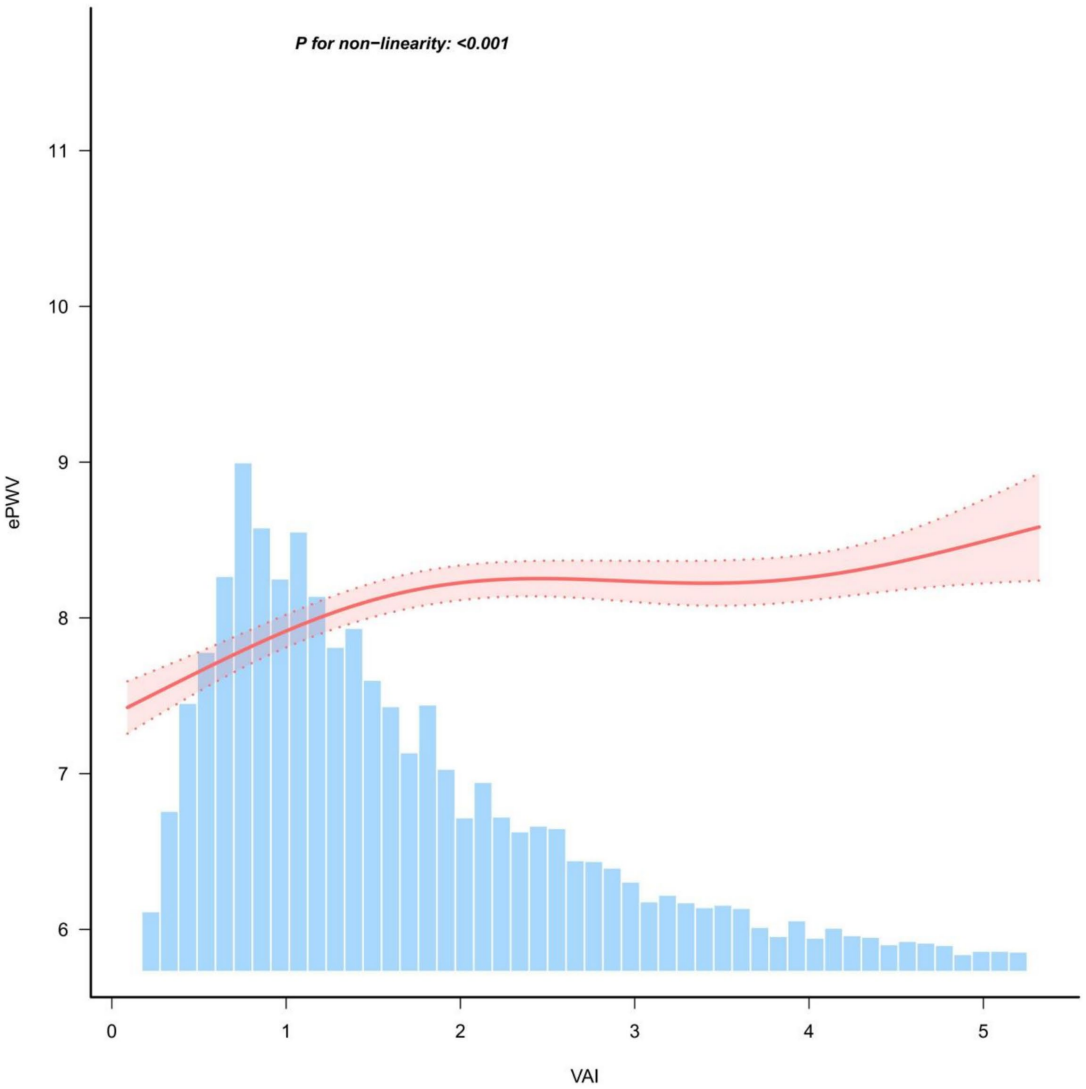
Restricted cubic spline analysis of the relationship between VAI and ePWV.

Restricted cubic spline models were constructed to fit the curve between VAI and ePWV. Smoothed curve fits between variables are indicated by the solid red line, and shaded bands indicate 95% confidence intervals. Adjustment for covariates revealed a non-linear relationship between VAI values and ePWV (*P* for non-linearity <0.001) (Figure 2).

**Figure 3:**
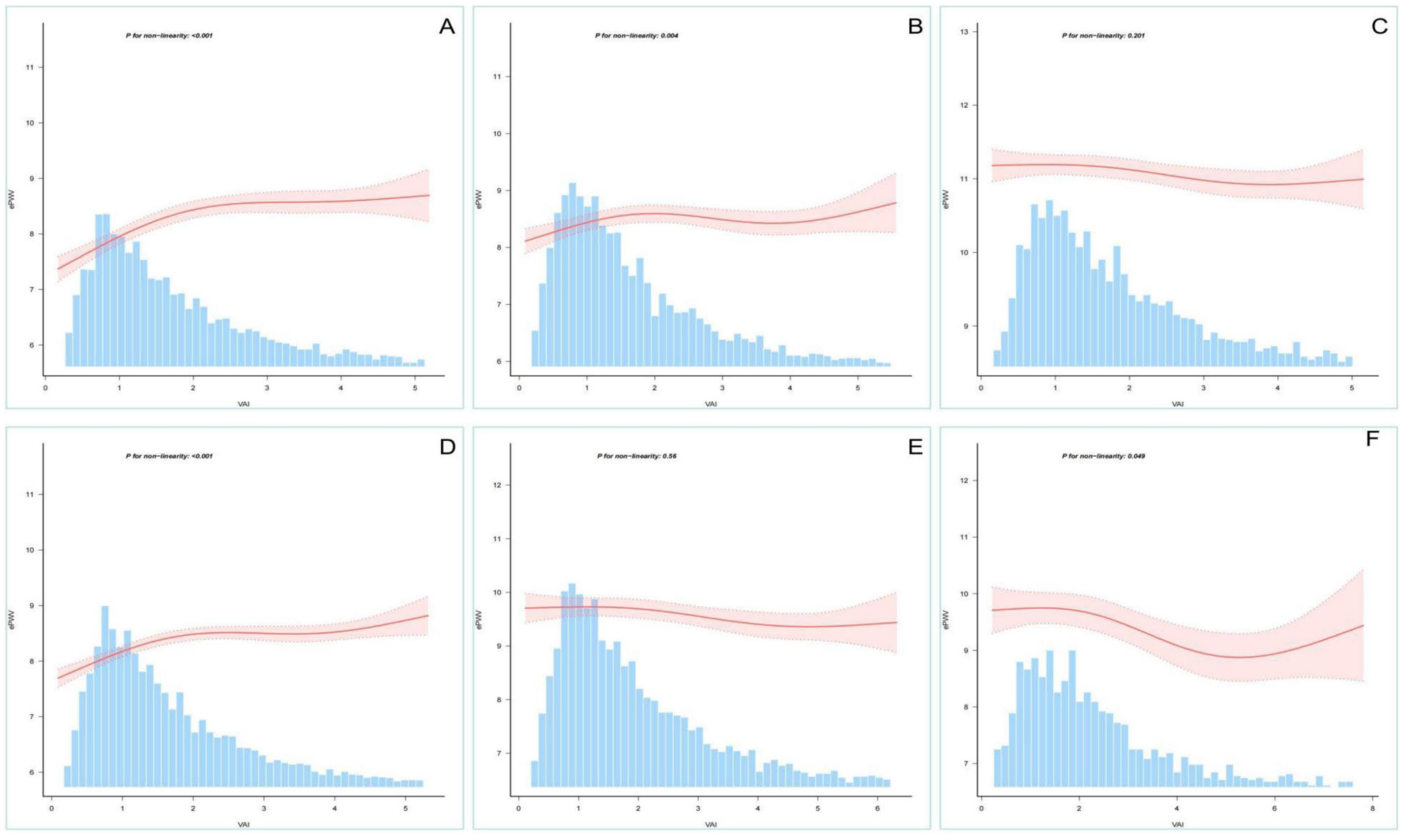
Restricted cubic spline analysis stratified by age, sex, etc. Association between VAI and ePWV stratified by (**A**: men; **B**: women), age (**C**: >60 years; **D**: <60 years) and special category (**E**: hypertension; **F**: diabetes).

To further validate the stability of the findings, we performed RCS analysis after stratifying the population. An “inverted L-shaped” non-linear relationship, consistent with the whole population, was observed in the male, female and age <60 years strata. The age >60 years, hypertension and diabetes strata showed higher levels of VAI and ePWV than the total population (Supplementary Table 3). Potential negative linear associations were found in stratified RCS analyses (Figure 3).

### Subgroup analysis and sensitivity analysis

**Figure 4:**
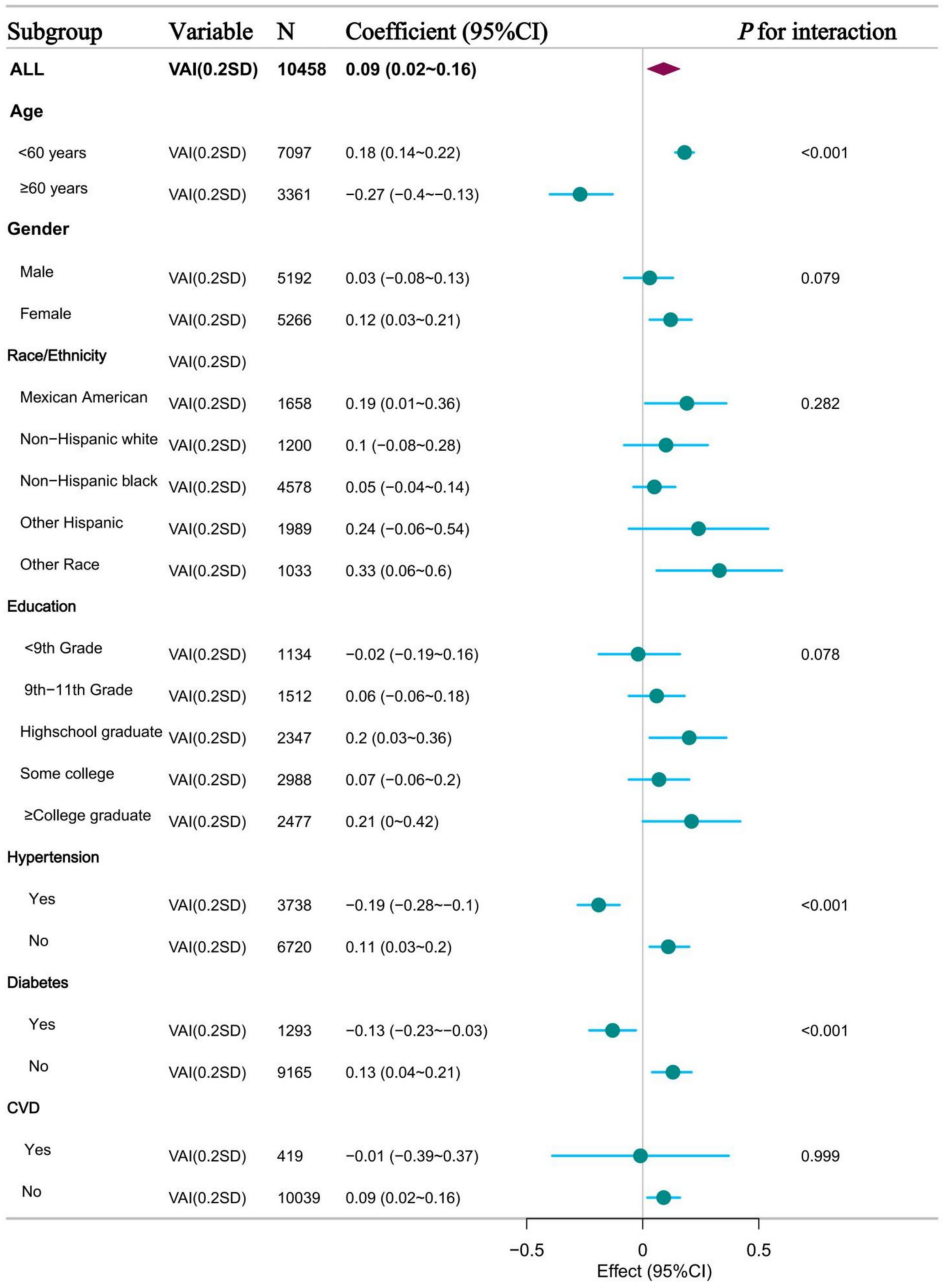
Forest plot of subgroup analysis of the relationship between VAI and ePWV.

To further validate the potential relationship between VAI and ePWV, we also analysed participants in subgroups based on age, sex, race, education, history of hypertension, history of diabetes, and history of CVD. Figure 4 shows the results of the subgroup analysis based on VAI quartiles, showing the relationship between VAI and ePWV in different subgroups. When VAI was grouped as a continuous and categorical variable, the results were consistent with the overall results in most subgroups. This means that there is a potential positive association between VAI and ePWV. However, a significant interaction was found in the subgroups of age > 60 years, hypertension and diabetes. This means that there was a possible negative association between VAI and ePWV (Supplementary Tables 4, 5).

### Inflection point analysis

Inflection point analysis In our study, we used restricted cubic spline analysis to explore the non-linear relationship between VAI and ePWV. The inflection point analysis showed that the relationship between VAI and ePWV changed significantly at a VAI value of 1.48. Specifically, when VAI was less than 1.48, there was a significant positive correlation between VAI and ePWV with a statistically significant difference (*p* < 0.001), whereas when VAI was greater than 1.48, this correlation was significantly attenuated and there was no statistically significant difference (*p* = 0.09) (Table 3 and Supplementary Figure 1).

**Table 3:** Inflection point analysis.

Model parameters	Breakpoint.Beta	Lower95ci	Upper95ci	*p* value
E_BK1	1.48	1.38	1.57	
Slope1	0.49	0.31	0.67	<0.001
Slope2	0.06	−0.01	0.12	0.09
Likelihood Ratio test			–	<0.001

Breakpoint.Beta: Breakpoint of the VAI value. Lower95ci: Lower limit of the 95% confidence interval. Upper95ci: Upper limit of the 95% confidence interval. Slope1: Effect size of the slope between VAI and ePWV for VAI values below the inflection point with statistically significant difference (*p* < 0.001). Slope2. Effect size of the slope between VAI and ePWV for VAI values above the inflection point with no statistically significant difference.

## Discussion

In this cross-sectional study, we examined the relationship between the VAI, a novel indicator of adiposity distribution and dysfunction, and ePWV, a marker of arterial stiffness, in a representative sample of US adults. Our results showed a significant positive association between VAI and ePWV, particularly below a VAI inflection point of 1.48. This association remained robust after adjustment for various confounders and was consistent across subgroup analyses, except for potential interactions within the age>60 years, hypertension, and diabetes subgroups. These findings suggest that abdominal obesity, as quantified by VAI, may be an important contributor to atherosclerosis. This study provides valuable insights into the complex interplay between obesity and vascular health and highlights the need for further research to develop targeted interventions to mitigate the impact of abdominal obesity on arterial stiffness and reduce cardiovascular risk.

Arterial stiffness, which progresses with age, results in thickening of the arterial walls and narrowing of the lumen (17). Arterial stiffness is largely determined by the severity of ischaemia in the affected organ, with most patients showing few clinical signs in the early stages. Research has shown that arterial stiffness is associated with an increased risk of cardiovascular disease, stroke and other diseases, yet a sensitive and specific laboratory diagnostic method for early arterial stiffness is lacking (18). As cf-PWV has not been widely used in clinical practice, ePWV has played a role in raising awareness of the risks of aortic stiffness and assisting physicians in its clinical application (19). The study involved using a previously published formula to assess ePWV and investigate its correlation with visceral adiposity index. Some recent research suggests a positive association between body fat levels and arterial stiffness, while other studies suggest a negative or no association (20). A study from the United States, which used VFA to accurately measure visceral fat to assess obesity, showed that the normal weight visceral obese group was associated with arterial stiffness and increased 10-year cardiovascular risk (21). A study from Spain, which also used body mass index, ratio and BMI to assess obesity, showed that indicators of obesity were inversely correlated with arterial stiffness, as measured by the cardio-ankle vascular index (CAVI) and brachial-ankle pulse wave velocity (22).

A study from Japan showed that measures of obesity, such as fat volume index, body mass index and body circumference index, were positively correlated with brachial-ankle PWV (23). In a study of Korean adults, ePWV was found to correlate more significantly with waist circumference than with BMI, supporting the concept that visceral obesity is more closely associated with arterial stiffness than total obesity (24). The results of our study regarding the association between visceral adiposity index and vascular stiffness are consistent with this. Therefore, our study offers the following advantages: The first notable advantage is that ePWV, as a marker of arterial stiffness, is more accessible and easily reproducible in clinical practice; the second important feature is that VAI, as a measure of visceral adipose function, is a sex-specific index that includes simple anthropometric and common epidemiological parameters, and this calculated value provides an association between abdominal obesity and arterial stiffness; Thirdly, the study we conducted this time had a sufficiently large sample size; and fourthly, for clinical purposes, this association provides a simple, valid and comprehensive assessment of cardiovascular disease in primary prevention populations and even in Stage 0 prevention groups, which is crucial for reducing the global burden of cardiovascular events. Future clinical guidelines and public health policies on obesity and cardiovascular health may be influenced by the results of this study.

A number of underlying mechanisms have been proposed to elucidate the initiation and progression of obesity and arterial stiffness. On the one hand, visceral adiposity actively secretes large amounts of inflammatory mediators, which can lead to increased insulin resistance, enhanced oxidative stress and endothelial cell dysfunction, all of which induce structural changes in the arterial wall and thus promote arterial stiffness (25). A critical element to consider is the role of adipokines, bioactive peptides with endocrine activity released from visceral adipose tissue. For example, leptin, an adipokine, is thought to activate the sympathetic nervous system. This activation can lead to increased blood pressure and increased arterial stiffness (26). On the other hand, levels of lipocalin, which has anti-inflammatory and anti-atherosclerotic properties, tend to be reduced in individuals with visceral obesity (27). It is also noteworthy that visceral adipose tissue actively expresses and releases components of the renin-angiotensin system, such as angiotensinogen and angiotensin II. The involvement of this system in the development of hypertension and vascular remodelling is well established, and both play an important role in atherosclerosis (28). In addition, abdominal obesity and atherosclerosis share common risk factors, including hypertension, diabetes, dyslipidaemia and sedentary lifestyle. It is conceivable that these diseases, while increasing atherosclerosis, may also have feedback mechanisms that indirectly contribute to abdominal obesity (29, 30).

In the present study, we found a significant positive correlation between VAI and ePWV. However, the basal level of ePWV was significantly higher than the total population level in the subgroups of patients older than 60 years, hypertensive patients and diabetic patients (Supplementary Table 6). An interaction between VAI and ePWV was observed in these subgroups. This phenomenon suggests that obesity seems to have an “inverse effect” on atherosclerosis in these specific subgroups. The higher the degree of VAI, the less pronounced the increase in ePWV compared to the total population. This phenomenon may be related to the interaction of several pathophysiological mechanisms associated with VAI.

The relationship between visceral obesity and arterial stiffness is not a simple linear one. Visceral obesity has been shown to be strongly associated with increased arterial stiffness. VAT secretes a variety of inflammatory factors and adipokines, such as leptin and lipocalin. These factors contribute to increased arterial stiffness by affecting insulin resistance, oxidative stress and endothelial cell function, promoting structural changes in the arterial wall (26) However, in specific populations, visceral obesity may have a complex effect on arterial stiffness through other mechanisms. In the subgroup aged &gt;60 years, ePWV levels were significantly higher than in the total population. This is consistent with previous studies showing that atherosclerosis increases with age (19). However, the positive correlation between VAI and ePWV was attenuated and even reversed in this subgroup. This phenomenon may be related to the pathophysiological mechanisms of atherosclerosis in the elderly population. With age, the ratio of collagen to elastin in the arterial wall changes, leading to increased arterial stiffness (7). In this context, obesity may have a “buffering” effect on arterial stiffness by affecting the metabolic and inflammatory state of the vascular wall. In addition, there is a high prevalence of comorbid chronic diseases (e.g., hypertension, hyperuricemia,diabetes mellitus) in the elderly population, and these diseases may themselves exacerbate arterial stiffness, thus masking the direct effect of obesity on arterial stiffness (25, 31). In the hypertensive subgroup, ePWV values were also significantly higher than in the total population. Hypertension is a major risk factor for increased atherosclerosis. It can lead to structural remodelling of the arterial wall and increased stiffness by increasing blood flow shear and vessel wall pressure (17). In this subgroup, the interaction between VAI and ePWV may be related to the effect of obesity on blood pressure regulation. Obese patients tend to have hyperactivation of the sympathetic nervous system, which may further exacerbate elevated blood pressure and increased arterial stiffness in hypertensive patients (20). However, obesity may also have a complex effect on arterial stiffness in hypertensive patients by affecting the renin-angiotensin-aldosterone system (RAAS) and vascular endothelial function. On the one hand, obesity may exacerbate arterial stiffness by increasing RAAS activation; on the other hand, obesity may have a protective effect on arterial stiffness by affecting vascular endothelial function (27). In the diabetic subgroup, ePWV values were significantly higher than in the total population, which is consistent with previous studies indicating the presence of higher arterial stiffness in diabetic patients (13). Patients with diabetes tend to have insulin resistance, hyperglycaemia and a chronic inflammatory state. Together, these factors lead to increased glycosylation and oxidative stress in the arterial wall, exacerbating atherosclerosis (29). In this subgroup, the interaction between VAI and ePWV may be related to the effect of obesity on the metabolic status of diabetic patients. Obese patients tend to have more severe insulin resistance, which may further exacerbate elevated blood glucose and increased arterial stiffness in diabetic patients (11). However, obesity may also have some protective effect on atherosclerosis by affecting lipid metabolism and inflammatory status in diabetic patients. For example, obese patients tend to have higher levels of high-density lipoprotein cholesterol (HDL-C), which may have a protective effect on oxidative stress and the inflammatory state of the arterial wall.

### Limitations

We acknowledge several limitations of our study. First, in the cross-sectional analyses, we excluded data with missing exposures and outcomes, which may introduce selection bias. Second, some of the data were collected by questionnaire, which may have introduced significant recall bias. Despite accounting for various confounding factors, there may still be other yet-to-be-discovered factors that could influence the results of the study and thus affect the validity of the findings. Further rigorously designed clinical trials are needed to investigate the potential association between visceral adiposity index and ePWV. In addition, the cross-sectional design of our study did not allow us to establish a causal relationship between VAI and ePWV.

### Conclusion

In this cross-sectional study, a significant positive association was observed between visceral adiposity index and estimated pulse wave velocity in a representative sample of US adults, particularly below a VAI inflection point of 1.48. This association remained robust after adjustment for various confounders and was consistent across subgroup analyses, highlighting the potential role of abdominal obesity in atherosclerosis and the importance of addressing this issue to reduce cardiovascular risk. Further research is warranted to explore interventions that may mitigate the effects of abdominal obesity on atherosclerosis.

## Data Availability

Publicly available datasets were analyzed in this study. This data can be found here: All the raw data required in this study can be extracted from the NHANES online website. For further inquiries, please contact the first author or corresponding author.

## References

[1] Wang H Yu X Guo J Ma S Liu Y Hu Y. Burden of cardiovascular disease among the Western Pacific region and its association with human resources for health, 1990-2021: a systematic analysis of the global burden of disease study 2021. Lancet Reg Health West Pac. (2024) 51:101195. doi: 10.1016/j.lanwpc.2024.101195, PMID: 39286450 PMC11404088

[2] Lee J Song RJ Musa Yola I Shrout TA Mitchell GF Vasan RS. Association of Estimated Cardiorespiratory Fitness in midlife with Cardiometabolic outcomes and mortality. JAMA Netw Open. (2021) 4:e2131284. doi: 10.1001/jamanetworkopen.2021.31284, PMID: 34714339 PMC8556623

[3] Mengi S Januzzi JL Jr Cavalcante JL Avvedimento M Galhardo A Bernier M. Aortic stenosis, heart failure, and aortic valve replacement. JAMA Cardiol. (2024) 9:1159–68. doi: 10.1001/jamacardio.2024.3486, PMID: 39412797

[4] Dangardt F Charakida M Georgiopoulos G Chiesa ST Rapala A Wade KH. Association between fat mass through adolescence and arterial stiffness: a population-based study from the Avon longitudinal study of parents and children. Lancet Child Adolesc Health. (2019) 3:474–81. doi: 10.1016/S2352-4642(19)30105-1, PMID: 31126896 PMC6558973

[5] Fan Y Yang S Ruan L Zhang C Cao M. Association between life's essential 8 and estimated pulse wave velocity among adults in the US: a cross-sectional study of NHANES 2011-2018. Front Public Health. (2024) 12:1388424. doi: 10.3389/fpubh.2024.1388424, PMID: 38873301 PMC11169870

[6] Covic A Siriopol D. Pulse wave velocity ratio: the new "gold standard" for measuring arterial stiffness. Hypertension. (2015) 65:289–90. doi: 10.1161/HYPERTENSIONAHA.114.04678, PMID: 25452468

[7] Marshall AG Neikirk K Afolabi J Mwesigwa N Shao B Kirabo A. Update on the use of pulse wave velocity to measure age-related vascular changes. Curr Hypertens Rep. (2024) 26:131–40. doi: 10.1007/s11906-023-01285-x, PMID: 38159167 PMC10955453

[8] Huang H Bu X Pan H Yang S Cheng W Shubhra QTH. Estimated pulse wave velocity is associated with all-cause and cardio-cerebrovascular disease mortality in stroke population: results from NHANES (2003-2014). Front Cardiovasc Med. (2023) 10:1140160. doi: 10.3389/fcvm.2023.1140160, PMID: 37153456 PMC10154635

[9] Shi Y Yu C Zhou W Wang T Zhu L Bao H. Estimated pulse wave velocity as a predictor of all-cause and cardiovascular mortality in patients with hypertension in China: a prospective cohort study. Front Cardiovasc Med. (2024) 11:1365344. doi: 10.3389/fcvm.2024.1365344, PMID: 38742177 PMC11089216

[10] Zhou XD Chen QF Yang W Zuluaga M Targher G Byrne CD. Burden of disease attributable to high body mass index: an analysis of data from the global burden of disease study 2021. EClinicalMedicine. (2024) 76:102848. doi: 10.1016/j.eclinm.2024.102848, PMID: 39386160 PMC11462227

[11] Zou X Zhou X Li Y Huang Q Ni Y Zhang R. Gender-specific data-driven adiposity subtypes using deep-learning-based abdominal CT segmentation. Obesity (Silver Spring). (2023) 31:1600–9. doi: 10.1002/oby.23741, PMID: 37157112

[12] Yaskolka Meir A Keller M Bernhart SH Rinott E Tsaban G Zelicha H. Lifestyle weight-loss intervention may attenuate methylation aging: the CENTRAL MRI randomized controlled trial. Clin Epigenetics. (2021) 13:48. doi: 10.1186/s13148-021-01038-0, PMID: 33663610 PMC7934393

[13] Liu C Pan H Kong F Yang S Shubhra QTH Li D. Association of arterial stiffness with all-cause and cause-specific mortality in the diabetic population: a national cohort study. Front Endocrinol (Lausanne). (2023) 14:1145914. doi: 10.3389/fendo.2023.1145914, PMID: 36967807 PMC10031114

[14] Amato MC Giordano C Galia M Criscimanna A Vitabile S Midiri M. Visceral adiposity index: a reliable indicator of visceral fat function associated with cardiometabolic risk. Diabetes Care. (2010) 33:920–2. doi: 10.2337/dc09-1825, PMID: 20067971 PMC2845052

[15] Zierfuss B Höbaus C Herz CT Pesau G Koppensteiner R Schernthaner GH. Predictive power of novel and established obesity indices for outcome in PAD during a five-year follow-up. Nutr Metab Cardiovasc Dis. (2020) 30:1179–87. doi: 10.1016/j.numecd.2020.03.019, PMID: 32451274

[16] Greve SV Laurent S Olsen MH. Estimated pulse wave velocity calculated from age and mean arterial blood pressure. Pulse (Basel). (2017) 4:175–9. doi: 10.1159/000453073, PMID: 28229052 PMC5290427

[17] d'El-Rei J Cunha MR Mattos SDS Marques BC Menezes VPD Cunha AR. Microvascular reactivity in hypertensive patients with high body adiposity. Arq Bras Cardiol. (2020) 115:896–904. doi: 10.36660/abc.20190364, PMID: 33295453 PMC8452213

[18] Ataee Z Aghaee A Sobhani SR Ebrahimi Miandehi E Pirzadeh P Alinezhad-Namaghi M. Evaluation of arterial stiffness and its relation to innovative anthropometric indices in Persian adults. Int J Hypertens. (2023) 2023:2180923. doi: 10.1155/2023/2180923, PMID: 36726690 PMC9886491

[19] Vishram-Nielsen JKK Laurent S Nilsson PM Linneberg A Sehested TSG Greve SV. Does estimated pulse wave velocity add prognostic information?: MORGAM prospective cohort project. Hypertension. (2020) 75:1420–8. doi: 10.1161/HYPERTENSIONAHA.119.14088, PMID: 32275189

[20] Gong J Han Y Gao G Chen A Fang Z Lin D. Sex-specific difference in the relationship between body fat percentage and arterial stiffness: results from Fuzhou study. J Clin Hypertens (Greenwich). (2023) 25:286–94. doi: 10.1111/jch.14649, PMID: 36815754 PMC9994159

[21] Zheng L Sun A Han S Qi R Wang R Gong X. Association between visceral obesity and 10-year risk of first atherosclerotic cardiovascular diseases events among American adults: National Health and nutrition examination survey. Front Cardiovasc Med. (2023) 10:1249401. doi: 10.3389/fcvm.2023.1249401, PMID: 37674809 PMC10479018

[22] Saz-Lara A Cavero-Redondo I Moreno-Herráiz N Rescalvo-Fernández E Berlanga-Macías C Medrano M. Association between body shape index and arterial stiffness: results of the EVasCu study and a meta-analysis. Int J Obes. 49:554–63. doi: 10.1038/s41366-024-01663-8, PMID: 39468316

[23] Yoshida Y Nakanishi K Jin Z Daimon M Ishiwata J Sawada N. Association between progression of arterial stiffness and left ventricular remodeling in a community-based cohort. JACC Adv. (2023) 2:100409. doi: 10.1016/j.jacadv.2023.100409, PMID: 38938996 PMC11198086

[24] Kim HL Joh HS Lim WH Seo JB Kim SH Zo JH. Associations of estimated pulse wave velocity with body mass index and waist circumference among general Korean adults. Meta. (2023) 13:1082. doi: 10.3390/metabo13101082, PMID: 37887407 PMC10608635

[25] Ardissino M McCracken C Bard A Antoniades C Neubauer S Harvey NC. Pericardial adiposity is independently linked to adverse cardiovascular phenotypes: a CMR study of 42 598 UK biobank participants. Eur Heart J Cardiovasc Imaging. (2022) 23:1471–81. doi: 10.1093/ehjci/jeac101, PMID: 35640889 PMC9584621

[26] Engin A. Endothelial dysfunction in obesity and therapeutic targets. Adv Exp Med Biol. (2024) 1460:489–538. doi: 10.1007/978-3-031-63657-8_17, PMID: 39287863

[27] Opdebeeck B Huysmans I Van den Branden A Orriss IR D'Haese PC Verhulst A. Deletion of the P2Y(2) receptor aggravates internal elastic lamina calcification in chronic kidney disease mice through upregulation of alkaline phosphatase and lipocalin-2. FASEB J. (2023) 37:e22701. doi: 10.1096/fj.202201044R 36520031

[28] Takeda Y Demura M Yoneda T Takeda Y. DNA methylation of the angiotensinogen gene, AGT, and the aldosterone synthase gene, CYP11B2 in cardiovascular diseases. Int J Mol Sci. (2021) 22:4587. doi: 10.3390/ijms22094587, PMID: 33925539 PMC8123855

[29] Zheng J Hu Y Xu H Lei Y Zhang J Zheng Q. Normal-weight visceral obesity promotes a higher 10-year atherosclerotic cardiovascular disease risk in patients with type 2 diabetes mellitus-a multicenter study in China. Cardiovasc Diabetol. (2023) 22:137. doi: 10.1186/s12933-023-01876-7, PMID: 37308932 PMC10262529

[30] West HW Siddique M Williams MC Volpe L Desai R Lyasheva M. Deep-learning for Epicardial adipose tissue assessment with computed tomography: implications for cardiovascular risk prediction. JACC Cardiovasc Imaging. (2023) 16:800–16. doi: 10.1016/j.jcmg.2022.11.018, PMID: 36881425 PMC10663979

[31] Cassano V Crescibene D Hribal ML Pelaia C Armentaro G Magurno M. Uric acid and vascular damage in essential hypertension: role of insulin resistance. Nutrients. (2020) 12:2509. doi: 10.3390/nu12092509, PMID: 32825165 PMC7551393

