# Supplementary material for: The relationship between visceral adiposity index and estimated pulse wave velocity: insights from NHANES database
Source: Front Nutr. 2025 Jun 11;12:1544084. doi: 10.3389/fnut.2025.1544084 (PMC12189020; doi:10.3389/fnut.2025.1544084)
Supplement: Supplementary file 1 [file Data_Sheet_1.zip › Supplementary material/Supplementary table1 Linear univariate regression.docx]

**Supplementary table 1** Linear univariate regression analysis between and ePWV concentration.

| **Item** | **Coeff.(95%CI)** | **P(t-test)** |
| --- | --- | --- |
| Age (Year) | 0.11 (0.11,0.11) | < 0.001 |
| Gender: Female vs Male | -0.26 (-0.35,-0.18) | < 0.001 |
| Race/Ethnicity,ref.=Mexican American |  |  |
| Non-Hispanic white | 0.33 (0.17,0.49) | < 0.001 |
| Non-Hispanic black | 0.75 (0.63,0.87) | < 0.001 |
| Other Hispanic | 0.46 (0.32,0.6) | < 0.001 |
| Other Race | -0.15 (-0.32,0.02) | 0.08 |
| Education: ref.<9th Grade |  |  |
| 9th-11th Grade | -0.82(-0.99,-0.66) | < 0.001 |
| High school graduate | -0.7 (-0.85,-0.55) | < 0.001 |
| Some college | -1.05 (-1.19,-0.9) | < 0.001 |
| ≥College graduate | -1.12(-1.27,-0.97) | < 0.001 |
| Marital: No vs Yes | -0.05 (-0.13,0.04) | 0.296 |
| PIR: ref.=Low income（<1.3） |  | < 0.001 |
| Medium（1.3-3.5） | 0.37 (0.26,0.47) |  |
| High income（ ≥3.5） | 0.21 (0.1,0.32) |  |
| Energy(kcal) | 0 (0,0) | < 0.001 |
| Protein (gm) | -0.01 (-0.01,-0.01) | < 0.001 |
| Carbohydrate (gm) | 0 (0,0) | < 0.001 |
| Total sugars(gm) | 0 (0,0) | < 0.001 |
| Dietary fiber(gm) | -0.01 (-0.01,0) | < 0.001 |
| Total fat(gm) | -0.01 (-0.01,-0.01) | < 0.001 |
| BMI, (Kg/M2) | 0.02 (0.01,0.02) | < 0.001 |
| Wait circumference,(cm) | 0.03 (0.02,0.03) | < 0.001 |
| SBP, mmHg | 0.08 (0.08,0.08) | < 0.001 |
| DBP, mmHg | 0.03 (0.02,0.03) | < 0.001 |
| TG,mmol/L | 0.1 (0.06,0.13) | < 0.001 |
| LDL-cholesterol ,mmol/L | 0.03 (-0.01,0.08) | 0.18 |
| HDL-Cholesterol ,mmol/L | 0.33 (0.23,0.43) | < 0.001 |
| FPG,mmol/L | 0.23 (0.21,0.26) | < 0.001 |
| Hemoglobin A1c (%) | 0.5 (0.46,0.53) | < 0.001 |
| Hypertension,No vs Yes | 2.06 (1.99,2.14) | < 0.001 |
| Diabetes, No vs Yes | 1.62 (1.49,1.74) | < 0.001 |
| CVD, No vs Yes | 2.29 (2.09,2.5) | < 0.001 |
| MAP , mmHg | 0.09 (0.09,0.09) | < 0.001 |
| VAI (0.2SD) | 0.1 (0.04,0.17) | 0.003 |

**Abbreviations:** BMI, body mass index; PIR, family poverty income ratio; SBP,systolic blood pressure;DBP: diastolic blood pressure;, FBG: fasting blood glucose;TG,Triglyceride;CVD, Cardiovascular disease; MAP,mean arterial pressure;ePWV,Estimated pulse wave velocity.
