# Supplementary material for: The relationship between visceral adiposity index and estimated pulse wave velocity: insights from NHANES database
Source: Front Nutr. 2025 Jun 11;12:1544084. doi: 10.3389/fnut.2025.1544084 (PMC12189020; doi:10.3389/fnut.2025.1544084)
Supplement: Supplementary file 1 [file Data_Sheet_1.zip › Supplementary material/Supplementary table2 Covariate screening.docx]

**Supplementary table2**  Covariate screening by calculating the variance inflation factor VIF

| **Term1** | **Coeff1** | **Change.**  **percentage1** | **Term2** | **Coeff2** | **Change.**  **percentage2** | **GVIF** | **DF** | **GVIF^(1/(2*Df))** | **Colinearity** | **Select** | **Select.VIF** |
| --- | --- | --- | --- | --- | --- | --- | --- | --- | --- | --- | --- |
| Crude | 0.02 | Ref. | Full | -0.06 | Ref. | 10.442 | 1 | 3.231 | 1 | Ref. | Pending |
| Blood glucose | -0.01 | -143.6 | Blood glucose | -0.07 | 5.6 | 1.532 | 1 | 1.238 | 0 | Yes | Yes |
| Gender | 0.02 | 0.4 | Gender | -0.07 | 10.7 | 2.181 | 1 | 1.477 | 0 | Yes | Yes |
| Age | 0 | -87.1 | Age | -0.15 | 134.4 | 1.993 | 1 | 1.412 | 0 | Yes | Yes |
| Race/Ethnicity | 0.02 | -3.2 | Race/Ethnicity | -0.06 | -2.8 | 1.655 | 4 | 1.065 | 0 | No | No |
| Education | 0.01 | -32.2 | Education | -0.07 | 2.3 | 1.679 | 4 | 1.067 | 0 | Yes | Yes |
| Marital Status | 0.02 | -0.7 | Marital Status | -0.06 | 0.7 | 1.095 | 1 | 1.046 | 0 | No | No |
| PIR | 0.03 | 25.8 | PIR | -0.06 | -6.5 | 1.417 | 1 | 1.19 | 0 | Yes | Yes |
| Energy | 0.02 | 7.5 | Energy | -0.06 | 0.4 | 34.689 | 1 | 5.89 | 1 | No | Pending |
| Protein | 0.02 | 4.5 | Protein | -0.06 | -0.2 | 3.764 | 1 | 1.94 | 0 | No | No |
| Carbohydrate | 0.02 | 13.3 | Carbohydrate | -0.07 | 1.5 | 21.564 | 1 | 4.644 | 1 | Yes | Pending |
| Total sugars | 0.02 | 15 | Total sugars | -0.06 | 0.3 | 5.398 | 1 | 2.323 | 1 | Yes | Pending |
| Dietary fibe | 0.02 | -0.2 | Dietary fibe | -0.06 | -0.1 | 2.188 | 1 | 1.479 | 0 | No | No |
| Total fat | 0.02 | -1.6 | Total fat | -0.06 | -0.3 | 8.461 | 1 | 2.909 | 1 | No | Pending |
| BMI | 0.02 | -22 | BMI | -0.05 | -24.1 | 8.062 | 1 | 2.839 | 1 | Yes | Pending |
| Wait circumference | -0.01 | -125.4 | Wait circumference | -0.06 | -6.9 | 8.796 | 1 | 2.966 | 1 | Yes | Pending |
| Hypertension | -0.01 | -137.8 | Hypertension | -0.06 | 0 | 1.449 | 1 | 1.204 | 0 | Yes | Yes |
| Diabetes | 0 | -86.5 | Diabetes | -0.06 | -0.6 | 1.579 | 1 | 1.256 | 0 | Yes | Yes |
| CVD | 0.02 | -18 | CVD | -0.06 | -0.7 | 1.101 | 1 | 1.049 | 0 | Yes | Yes |
| SBP | -0.01 | -153.3 | SBP | -0.05 | -15.9 | 1.607 | 1 | 1.268 | 0 | Yes | Yes |
| DBP | 0.01 | -34.9 | DBP | -0.06 | -0.6 | 1.28 | 1 | 1.131 | 0 | Yes | Yes |
| TG | -0.1 | -585.9 | TG | -0.06 | 0.7 | 1623.45 | 1 | 40.292 | 1 | Yes | Pending |
| LDL-cholesterol | 0.12 | 483.5 | LDL-cholesterol | -0.01 | -81.4 | 11652.3 | 1 | 107.946 | 1 | Yes | Pending |
| TC | 0.01 | -57.6 | TC | -0.06 | 0.1 | 15261.5 | 1 | 123.538 | 1 | Yes | Pending |
| HDL-cholesterol | 0.05 | 114.8 | HDL-cholesterol | -0.06 | -0.1 | 2372.97 | 1 | 48.713 | 1 | Yes | Pending |

**Abbreviations:** PIR,Poverty income ratio; BMI,Body mass index;CVD,Cardiovascular disease; SBP,Systolic blood pressure;DBP: Diastolic blood pressure;TG,Triglyceride;TC,Total cholesterol;

HDL-cholesterol,high density lipoprotein-cholesterol;LDL-cholesterol,Low density lipoprotein-cholesterol.
