# Supplementary material for: The relationship between visceral adiposity index and estimated pulse wave velocity: insights from NHANES database
Source: Front Nutr. 2025 Jun 11;12:1544084. doi: 10.3389/fnut.2025.1544084 (PMC12189020; doi:10.3389/fnut.2025.1544084)
Supplement: Supplementary file 1 [file Data_Sheet_1.zip › Supplementary material/Supplementary Table3 Characterisation of VAI and ePWV stratified.docx]

**Supplementary Table 3**  Characterisation of VAI and ePWV stratified by hypertension, diabetes, CVD, sex and age.

|  | **Overall (n = 10458)** | **With Hypertension(n = 3738)** | **No Hypertension(n = 6720)** | ***P-Value*** |
| --- | --- | --- | --- | --- |
| ePWV, Median (IQR) | 8.3(6.1,10.5) | 9.6(7.5,11.7) | 7.6 (5.9,9.4) | < 0.001 |
| VAI, Median (IQR) | 1.4 (0.9, 2.4) | 1.7 (1.0, 2.8) | 1.3 (0.8, 2.2) | < 0.001 |
|  |  | **With Diabetes(n = 1293)** | **No Diabetes(n = 9165)** |  |
| ePWV, Median (IQR) | 8.3(6.1,10.5) | 9.7 (7.7,11.7) | 8.1 (6.0,10.2) | < 0.001 |
| VAI, Median (IQR) | 1.4 (0.9, 2.4) | 1.9 (1.2, 3.1) | 1.4 (0.8, 2.3) | < 0.001 |
|  |  | **With CVD(n = 419)** | **No CVD(n = 10039)** |  |
| ePWV, Median (IQR) | 8.3(6.1,10.5) | 9.6 (7.5,11.3) | 8.0 (6.2,10.3) | < 0.001 |
| VAI, Median (IQR) | 1.4 (0.9, 2.4) | 1.8 (1.1, 3.0) | 1.4 (0.7, 2.2) | 0.01 |
|  |  | **Age≥60(n = 3361)** | **Age<60(n = 7097)** |  |
| ePWV, Median (IQR) | 8.3(6.1,10.5) | 10.8 (9.3,12.1) | 7.1 (6.0,8.2) | < 0.001 |
| VAI, Median (IQR) | 1.4 (0.9, 2.4) | 1.5 (0.9, 2.5) | 1.4 (0.8, 2.4) | 0.776 |
|  |  | **Male (n = 5192)** | **Female(n = 5266)** |  |
| ePWV, Median (IQR) | 8.3(6.1,10.5) | 8.6 (6.9,11.3) | 8.3 (6.7,10.3) | 0.632 |
| VAI, Median (IQR) | 1.4 (0.9, 2.4) | 1.5 (1.2, 2.9) | 1.4 (0.8, 2.5) | 0.863 |

**Abbreviations:** CVD, Cardiovascular disease; ePWV,Estimated pulse wave velocity;VAIvisceral adiposity index
