# Supplementary material for: The relationship between visceral adiposity index and estimated pulse wave velocity: insights from NHANES database
Source: Front Nutr. 2025 Jun 11;12:1544084. doi: 10.3389/fnut.2025.1544084 (PMC12189020; doi:10.3389/fnut.2025.1544084)
Supplement: Supplementary file 1 [file Data_Sheet_1.zip › Supplementary material/Supplementary table4 Subgroup analysis .docx]

**Supplementary table4** Subgroup analysis when VAI is used as a continuous variable

| **Subgroup** | **Variable** | **N** | **Coefficient (95%CI)** | ***P* for interaction** |
| --- | --- | --- | --- | --- |
| **Age** |  |  |  |  |
| <60 | VAI(0.2SD) | 7097 | 0.18 (0.14~0.22) | <0.001 |
| ≥60 | VAI(0.2SD) | 3361 | -0.27 (-0.4~-0.13) |  |
| **Gender** |  |  |  |  |
| Male | VAI(0.2SD) | 5192 | 0.03 (-0.08~0.13) | 0.079 |
| Female | VAI(0.2SD) | 5266 | 0.12 (0.03~0.21) |  |
| **Race/Ethnicity** |  |  |  |  |
| Mexican American | VAI(0.2SD) | 1658 | 0.19 (0.01~0.36) | 0.282 |
| Non-Hispanic white | VAI(0.2SD) | 1200 | 0.1 (-0.08~0.28) |  |
| Non-Hispanic black | VAI(0.2SD) | 4578 | 0.05 (-0.04~0.14) |  |
| Other Hispanic | VAI(0.2SD) | 1989 | 0.24 (-0.06~0.54) |  |
| Other Race | VAI(0.2SD) | 1033 | 0.33 (0.06~0.6) |  |
| **Education** |  |  |  |  |
| <9th Grade | VAI(0.2SD) | 1134 | -0.02 (-0.19~0.16) | 0.078 |
| 9th-11th Grade | VAI(0.2SD) | 1512 | 0.06 (-0.06~0.18) |  |
| Highschool graduate | VAI(0.2SD) | 2347 | 0.2 (0.03~0.36) |  |
| Some college | VAI(0.2SD) | 2988 | 0.07 (-0.06~0.2) |  |
| ≥College graduate | VAI(0.2SD) | 2477 | 0.21 (0~0.42) |  |
| **Hypertension** |  |  |  |  |
| Yes | VAI(0.2SD) | 3738 | -0.19 (-0.28~-0.1) | <0.001 |
| No | VAI(0.2SD) | 6720 | 0.11 (0.03~0.2) |  |
| **Diabetes** |  |  |  |  |
| Yes | VAI(0.2SD) | 1293 | -0.13 (-0.23~-0.03） | <0.001 |
| No | VAI(0.2SD) | 9165 | 0.13 (0.04~0.21) |  |
| **Cardiovascular disease** |  |  |  |  |
| Yes | VAI(0.2SD) | 419 | -0.01 (-0.39~0.37) | 0.999 |
| No | VAI(0.2SD) | 10039 | 0.09 (0.02~0.16) |  |

**Abbreviations:**VAI(0.2SD),Visceral Adiposity Index with a standard deviation (SD) increment of 0.2.
