# Supplementary material for: The relationship between visceral adiposity index and estimated pulse wave velocity: insights from NHANES database
Source: Front Nutr. 2025 Jun 11;12:1544084. doi: 10.3389/fnut.2025.1544084 (PMC12189020; doi:10.3389/fnut.2025.1544084)
Supplement: Supplementary file 1 [file Data_Sheet_1.zip › Supplementary material/Supplementary table5 Subgroup analysis.docx]

**Supplementary table5** Subgroup analysis when using VAI as a categorical variable

| **Subgroup** | **Variable** | **N** | **Coefficient**  **Coefficient (95%CI)** | ***P* for interaction** |
| --- | --- | --- | --- | --- |
| **Age** |  |  |  | <0.001 |
| <60 | Q2 | 1814 | 0.21(0.14~0.28) |  |
|  | Q3 | 1692 | 0.40(0.33~0.48) |  |
|  | Q4 | 1724 | 0.62(0.55~0.7) |  |
| ≥60 | Q2 | 828 | 0.10(-0.05~0.26) |  |
|  | Q3 | 916 | -0.04（-0.2~0.11) |  |
|  | Q4 | 909 | -0.18(-0.04~-0.33) |  |
| **Gender** |  |  |  | <0.001 |
| Male | Q2 | 1292 | 0.25(0.10~0.41) |  |
|  | Q3 | 1254 | 0.34(0.18~0.50) |  |
|  | Q4 | 1278 | 0.19(0.04~0.35) |  |
| Female | Q2 | 1350 | 0.31(0.15~0.48) |  |
|  | Q3 | 1354 | 0.64(0.47~0.81) |  |
|  | Q4 | 1355 | 0.86(0.68~1.03) |  |
| **Race/Ethnicity** |  |  |  | 0.622 |
| Mexican American | Q2 | 384 | 0.37(0.07~0.66) |  |
|  | Q3 | 489 | 0.43(0.14~0.71) |  |
|  | Q4 | 527 | 0.54(0.25~0.82) |  |
| Non-Hispanic white | Q2 | 302 | 0.47(0.11~0.82) |  |
|  | Q3 | 332 | 0.88(0.52~1.23) |  |
|  | Q4 | 345 | 0.93(0.58~1.28) |  |
| Non-Hispanic black | Q2 | 1131 | 0.24(0.06~0.43) |  |
|  | Q3 | 1122 | 0.51(0.33~0.70) |  |
|  | Q4 | 1295 | 0.56(0.37~0.74) |  |
| Other Hispanic | Q2 | 569 | 0.23(0.01~0.44) |  |
|  | Q3 | 412 | 0.4(0.16~0.64) |  |
|  | Q4 | 237 | 0.3(0.01~0.59) |  |
| Other Race | Q2 | 256 | 0.27(-0.04~0.58) |  |
|  | Q3 | 253 | 0.45(0.13~0.76) |  |
|  | Q4 | 229 | 0.53(0.21~0.86) |  |
| **Education** |  |  |  | 0.107 |
| <9th Grade | Q2 | 254 | 0.46(0.02~0.89) |  |
|  | Q3 | 308 | 0.46(0.03~0.89) |  |
|  | Q4 | 390 | 0.57(0.16~0.99) |  |
| 9th-11th Grade | Q2 | 354 | 0.3(-0.02~0.61) |  |
|  | Q3 | 394 | 0.32(0~0.64) |  |
|  | Q4 | 444 | 0.34(0.02~0.65) |  |
| Highschool graduate | Q2 | 611 | 0.38(0.12~0.63) |  |
|  | Q3 | 603 | 0.67(0.41~0.93) |  |
|  | Q4 | 612 | 0.76(0.5~1.02) |  |
| Some college | Q2 | 733 | 0.28(0.07~0.48) |  |
|  | Q3 | 740 | 0.46(0.25~0.66) |  |
|  | Q4 | 764 | 0.55(0.34~0.76) |  |
| ≥College graduate | Q2 | 690 | 0.1(-0.1~0.29) |  |
|  | Q3 | 563 | 0.48(0.26~0.69) |  |
|  | Q4 | 423 | 0.37(0.14~0.6) |  |
| **Hypertension** |  |  |  | <0.001 |
| Yes | Q2 | 891 | 0.06(-0.14~0.27) |  |
|  | Q3 | 978 | 0.03(-0.18~0.23) |  |
|  | Q4 | 1189 | -0.27(-0.47~-0.07) |  |
| No | Q2 | 1751 | 0.13(0.01~0.25) |  |
|  | Q3 | 1630 | 0.33(0.21~0.45) |  |
|  | Q4 | 1444 | 0.4(0.27~0.52) |  |
| **Diabetes** |  |  |  | <0.001 |
| Yes | Q2 | 255 | 0.33(-0.05~0.71) |  |
|  | Q3 | 378 | 0.13(-0.23~0.49) |  |
|  | Q4 | 494 | -0.37(-0.72~-0.07) |  |
| No | Q2 | 2387 | 0.22(0.11~0.34) |  |
|  | Q3 | 2230 | 0.4(0.28~0.53) |  |
|  | Q4 | 2139 | 0.51(0.38~0.63) |  |
| **CVD** |  |  |  | 0.069 |
| Yes | Q2 | 90 | 0.13(-0.44~0.7) |  |
|  | Q3 | 103 | -0.24(-0.8~0.32) |  |
|  | Q4 | 145 | -0.19(-0.71~0.34) |  |
|  | Q3 | 103 | -0.24(-0.8~0.32) |  |
|  | Q4 | 145 | -0.19(-0.71~0.34) |  |
| No | Q2 | 2552 | 0.29(0.17~0.4) |  |
|  | Q3 | 2505 | 0.52(0.4~0.64) |  |
|  | Q4 | 2488 | 0.57(0.45~0.69) |  |

**Abbreviations: CVD,**Cardiovascular disease;Q2,Quartile2 (0.87-1.42);Q3,Quartile3 (1.42-2.41);Q4,Quartile4 (>2.41).
