# Supplementary material for: The relationship between visceral adiposity index and estimated pulse wave velocity: insights from NHANES database
Source: Front Nutr. 2025 Jun 11;12:1544084. doi: 10.3389/fnut.2025.1544084 (PMC12189020; doi:10.3389/fnut.2025.1544084)
Supplement: Supplementary file 1 [file Data_Sheet_1.zip › Supplementary material/Supplementary table6.docx]

**Supplementary table6** Comparison between VAI and ePWV grouped by age, hypertension and diabetes.

| **Age** | Total (n = 10458) | <60 (n = 7097) | ≥60 (n = 3361) | *P* |
| --- | --- | --- | --- | --- |
| VAI, Median (IQR) | 1.4 (0.9, 2.4) | 1.4 (0.8, 2.4) | 1.5 (0.9, 2.5) | < 0.001 |
| ePWV, Median (IQR) | 8.3 (6.1,10.7) | 7.1 (6.0,8.2) | 10.8 (9.3,12.1) | < 0.001 |
|  |  |  |  |  |
| **Hypertension** | Total (n = 10458) | No(n =6720） | Yes(n=3738） |  |
| VAI, Median (IQR) | 1.4 (0.9, 2.4) | 1.3 (0.8, 2.2) | 1.7 (1.0, 2.8) | < 0.001 |
| ePWV, Median (IQR) | 8.3 (6.1,10.7) | 7.6 (5.9,9.4) | 9.6(7.5,11.7) | < 0.001 |
|  |  |  |  |  |
| **Diabetes** | Total (n = 10458) | No(n=9165） | Yes(n=1293） |  |
| VAI, Median (IQR) | 1.4 (0.9, 2.4) | 1.4 (0.8, 2.3) | 1.9 (1.2, 3.1) | < 0.001 |
| ePWV, Median (IQR) | 8.3 (6.1,10.7) | 8.1 (6.0,10.2) | 9.7 (7.7,11.7) | < 0.001 |

**Abbreviations:**  ePWV,Estimated pulse wave velocity;VAIvisceral adiposity index;IQR,Interquartile Range.
