# Supplementary figures and images for: The relationship between visceral adiposity index and estimated pulse wave velocity: insights from NHANES database
Source: Front Nutr. 2025 Jun 11;12:1544084. doi: 10.3389/fnut.2025.1544084 (PMC12189020; doi:10.3389/fnut.2025.1544084)

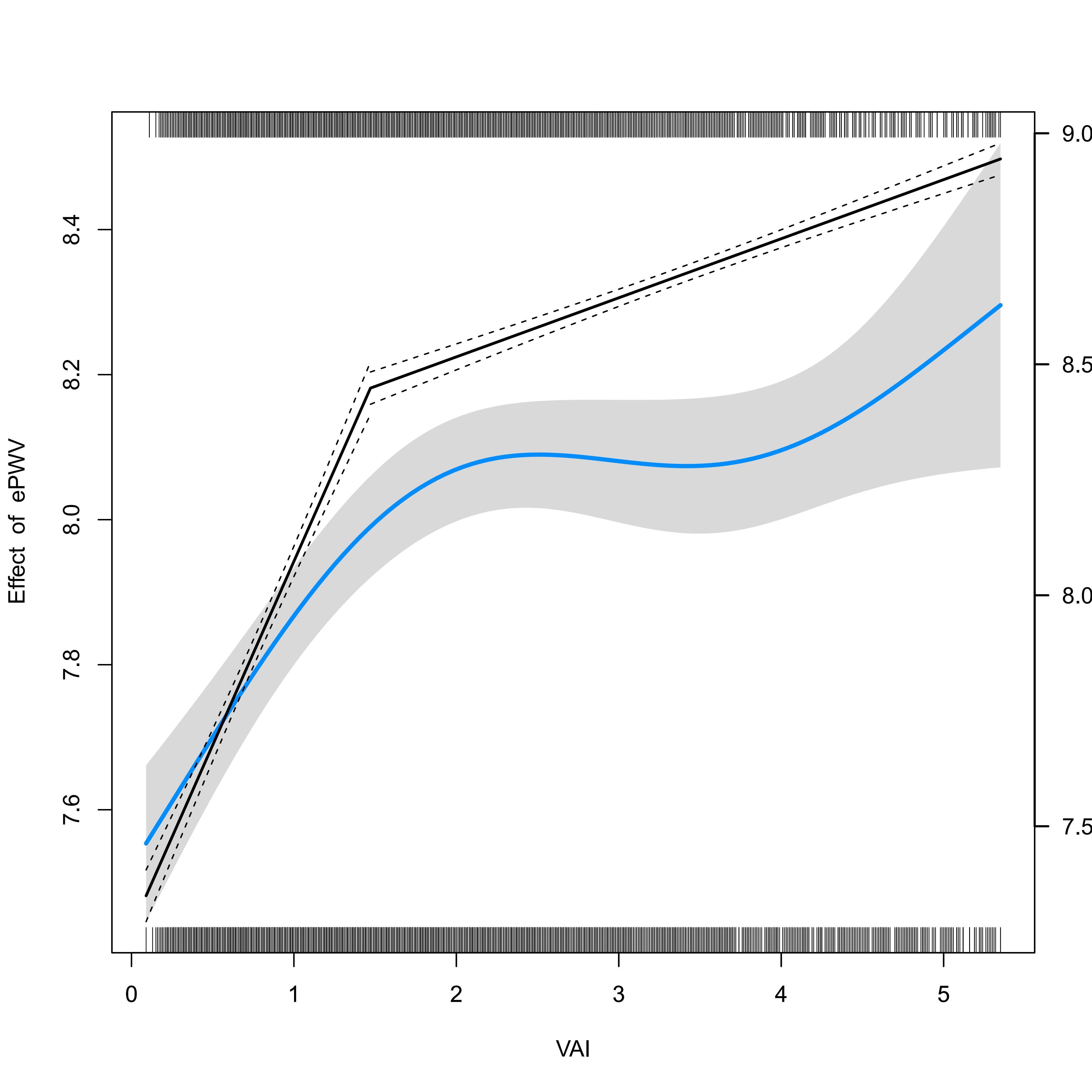

Supplement: Supplementary file 1 [file Data_Sheet_1.zip › Supplementary material/Supplementary figure1 300DPI.jpg]
